# Disruptive Innovation and Health Literacy

**DOI:** 10.3928/24748307-20180115-01

**Published:** 2018-02-07

**Authors:** Joseph M. Geskey

Limitations in health literacy have been projected to cost the United States economy between $106 billion and $238 billion annually (Vernon, Trujillo, Rosenbaum, & DeBuono, 2007), in addition to being associated with worse health care outcomes (Berkman, Sheridan, Donahue, Halpern & Crotty, 2011). Health literacy scores are independently associated with household income, and with lower scores associated with decreased income (Rikard, Thompson, McKinney, & Beauchamp, 2016). Unfortunately, people in the bottom 5th percentile of a country's income distribution have a life expectancy that is 25% shorter than those in the top 5th percentile (Cutler, Deaton, & Lleras-Muney, 2006). Therefore, it is critical for health care providers to understand the health effects of social and economic policies that affect not only individual people, but the communities in which they live (Marmot, 2005).

In 1962, the economist Gary Becker developed human capital theory, which is “concerned with activities that influence future real income through the imbedding of resources in people” (Becker, 1962). In 1972, Michael Grossman built upon Becker's work to develop the concept of health capital, whereby an individual's health depends on investing resources to increase its value (Grossman, 1972). Investing requires the deferment of present wants for an uncertain future distant benefit. Unfortunately, many people living in poverty expend a significant amount of their cognitive, economic, and physical capacities trying to manage housing, transportation, nutrition, and employment-related issues compared to more affluent people. Therefore, people who have constrained budgets, have to make difficult trade-offs to execute these tasks, and this can cause behavior control to be impaired because the decision-making process can deplete their willpower and self-control (Haushofer & Fehr, 2014).

Additionally, patients with limited health literacy can experience feelings of embarrassment and shame when interacting with health care providers and may try to hide or compensate for a lack of understanding (Baker et al., 1996). Many patients with chronic illness leave health care encounters with a different perception and recollection from health care professionals regarding issues that were discussed, decisions that were made, and the goals that were set (Parkin & Skinner, 2003). The problem of limited health literacy, particularly in disadvantaged socioeconomic groups with chronic diseases, begs for disruptive innovation to improve health care outcomes.

## What Is Disruptive Innovation in Health Care?

Disruptive innovation in health care occurs when service innovations that are cheaper, simpler, and more convenient are focused on the low end of the market because some of the more dominant players are focused on creating innovations for more profitable, high-end customers (Christensen, et al., 2000). Although using the terms “profit” and “customers” may raise justifiable concerns from health care providers, I am using Christenson's definition of disruptive innovation in the context of pursuing the “triple aim” of improving the experience of care, the health of populations, and reducing per capita health care costs. Berwick, Nolan, and Whittington (2008) highlight the possibility of disruptive innovation by advocating for the creation of an “integrator”; an entity that can serve people and families, strengthen primary care, deliver on the promise of population health, promote fiscally sound management, and integrate health care delivery across the continuum of care.

How does this translate to patients with limited health literacy who have challenging socioeconomic conditions? Health entities are currently focusing on constructing new buildings and offering more technologically advanced procedures and equipment to meet the needs of people who use consumer wearables to track health metrics and want the convenience of interacting with health care professionals via mobile apps and other technology. These offerings appeal to people who are more affluent, more technologically savvy, and are typically more profitable for health care systems, and the result is that many of the problems patients with limited health literacy experience are not being adequately addressed (Wynia & Osborn, 2010).

## Setting the Stage

The biology of aging, the impact of illness, and a person's baseline level of education are all important variables to consider when trying to effectively communicate information about illness, treatment options, and self-management skills. The inability to successfully execute these skills could have potentially life-limiting implications for patients, and yet our health care systems' educational efforts, whether in the inpatient or outpatient setting, don't receive the same degree of attention as other aspects of health care (Berkman, Sheridan, Donahue, Halpern, & Crotty, 2011; Wynia & Osborn, 2010).

Unfortunately, patients with limited health literacy who are not thriving in the “medicalized” portion of their health care can be labeled as “noncompliant” or a “frequent flier” if they happen to get hospitalized when others do just fine in an ambulatory setting. The term “noncompliant” is perhaps the most pejorative label in medicine, as creative, individualized solutions are no longer searched for by otherwise intuitive, empathetic health care professionals. Health care providers often react to patients' nonadherence in an ego-defensive manner (Heszen-Klemens, 1987), meaning that they feel it is not they who need to change how they manage the clinical encounter but rather the patients' unwillingness to listen and follow through with instructions that leads to suboptimal outcomes. Rather than blaming an individual patient, it is important to reframe the situation by internalizing what the quality movement in U.S. health care tries to promote—that poor outcomes are typically due to deficient processes rather than individual actions. This is where organizational approaches to health literacy can promote success by helping patients navigate the health care system, promote equity by reducing access barriers to disadvantaged populations, make health care information easier to understand, and promote the integration of health and social care (Annarumma & Palumbo, 2016),

Although organizational approaches to address health literacy can potentially improve patient experience and patient outcomes, between 95% and 99% of chronic disease care is still delivered by the individual (Funnell, 2000). Recently, neuroscientists have described a salience network in the brain that contributes to attention, motivation, and behavior (Shenhav et al., 2017). This explains why it is so hard to change behavior in people who direct most of their efforts toward daily survival, as the capacity of our brains to balance critical priorities is not unlimited. Therefore, there has to be a strategy in which patients, providers, and health systems can address limited health literacy in a holistic fashion.

## A Possible Solution

**Figure 1.**
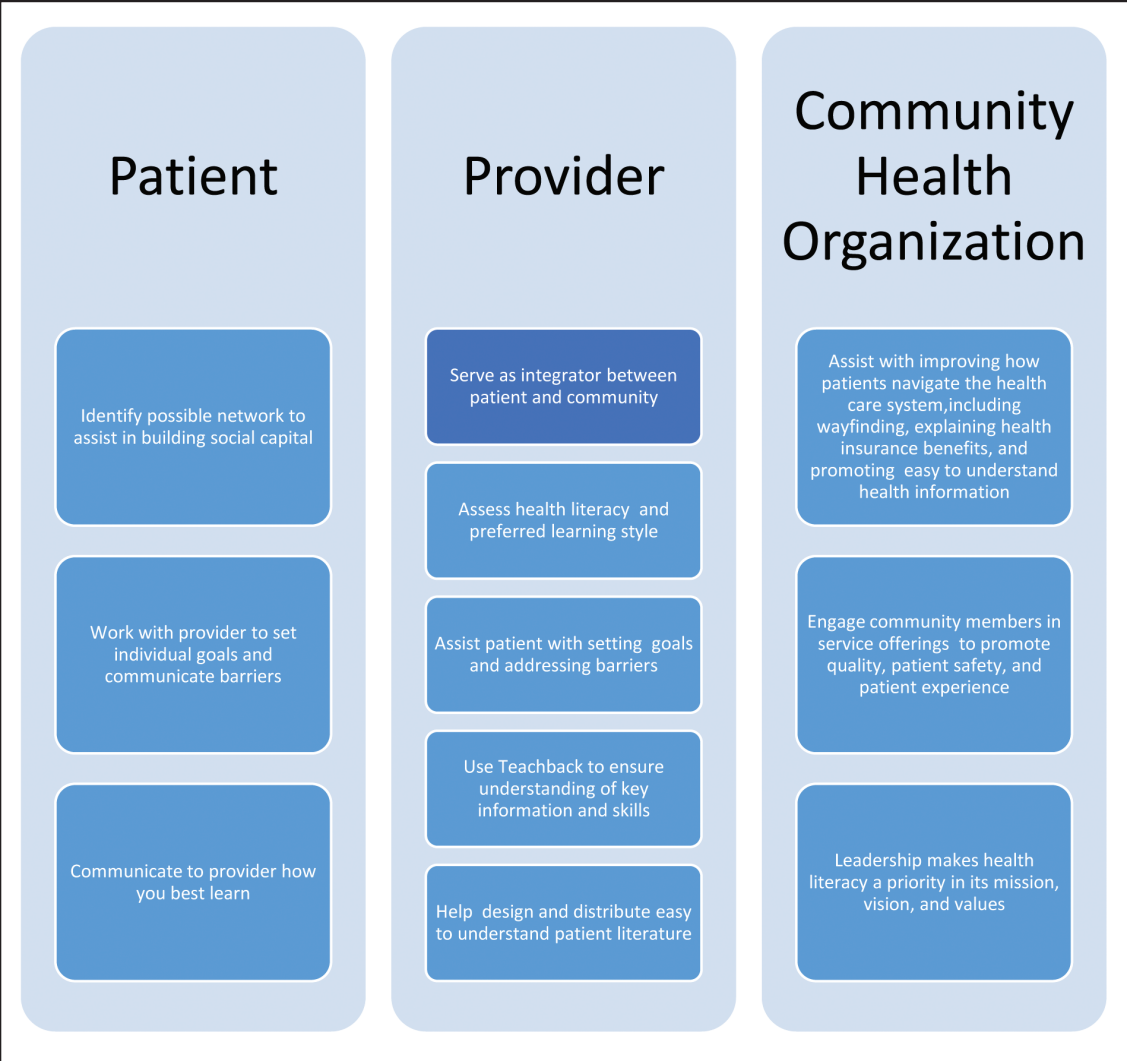
A patient–provider–community health organization approach to manage limited health literacy.

I have created a chronic disease curriculum predicated on formally measuring patients' health literacy and their level of activation. In eligible patients, myself and two home health nurses go into patients' homes for 1 hour a week for 4 weeks and deliver an educational curriculum based on their level of activation. All patients have been hospitalized with either a chronic obstructive pulmonary disease (COPD) exacerbation or congestive heart failure exacerbation, meet Medicare home-bound criteria, and have limited health literacy based on formal assessments. To date, we have visited more than 100 hundred patients in a geographic area that is below the state average for income and educational attainment and above the average for people living in poverty. Participation in the program to date has led to a 40% relative reduction in 30-day readmissions and improved engagement scores at the end of the program (Geskey, unpublished data). Visiting patient homes has allowed my team to know how patients live on a day-to-day basis, for who and what they live for, and how illness interferes with their goals and motivations. We ask every patient, “What is the one thing your illness has prevented you from doing that you like?” Every patient can immediately articulate the loss experienced due to chronic illness and can envision the confidence and joy achieving that goal again. We tell patients that we measure success not biomedically, but rather if they are able to achieve their goal(s). By spending time educating patients on a concept or skill in a manner which they learn best and ensuring that they can demonstrate and/or repeat this skill or concept, both the patient and provider can be assured they have demonstrated mastery that allows them to build self-efficacy so they can better manage their condition, communicate effectively to their primary care physician, and come closer to obtaining a patient-derived goal. For example, a man with COPD who is at the lowest level of activation doesn't fully understand the role he plays in his health care, so teaching him how to manage acute exacerbations of a chronic illness is beyond the scope of his capabilities. Our first step then would be to get him to make the connection that his COPD is influencing his ability to achieve his goal. From there, we can then go on to explain what COPD is, what his medications are for, and have him demonstrate how to appropriately use them, and then we can proactively problem-solve different scenarios so he can feel confident self-managing them. This approach helps foster trust among patients, providers, and health systems by building what Kim, Lim, & Park (2015) refer to as “bridging social capital,” which can modify the functional effect of low health literacy on health information resources, efficacy, and behaviors. Bridging social capital can improve overall human and health capital by allowing patients to use a network of support for various purposes in diverse situations that enhances their problem-solving abilities in regard to their health.**Figure 1** illustrates the integration of a patient-provider-community health organization in effectively managing patients with limited health literacy.

How can interventions like this be scaled to reach more people? Although technological solutions can support self-management and be offered to more people at a lower cost than home visits, not one of the patients I have visited at home uses a computer or a smart phone to research medical information, schedule appointments, or monitor test results. Proponents of technology-based solutions have to address the potential unanticipated consequences of having patients believe their conditions are too daunting to be self-managed, or conversely, give a false sense of security that it will keep them well in lieu of their active participation. In my opinion, technology certainly can play a positive role, but only after social capital has been built among patients, providers, and community health systems. Additionally, these offerings need to be designed and tested in patients with limited health literacy. A recent study in patients with type 2 diabetes demonstrated that a web-based design incorporating iterative user feedback and using a simple clear design and presentation that was interactive improved diabetes knowledge and intention to participate in physical activity (Muller et al., 2017). Additionally, incorporating the use of text messaging has been shown to be beneficial in patients with limited health literacy (Manganello et al., 2017). However, even as tech-savvy people age, the biology of aging in concert with advancing disease may inhibit the executive function necessary to navigate the complexity of chronic diseases, so just assuming that technology in health care will be more easily adopted in future generations may be a mistake.

In conclusion, primary care physicians working within a medical home model can serve an integrator function that assists patients with limited health literacy articulate a goal they would like to achieve while breaking down educational and communication barriers that prevent them from being successful. Moving forward, both physicians and their patients can use mutual experiences to help design and test the use of digital solutions that help meet future patients' needs on a broader scale.
